# Autophagy Drives Galectin-1 Secretion From Tumor-Associated Macrophages Facilitating Hepatocellular Carcinoma Progression

**DOI:** 10.3389/fcell.2021.741820

**Published:** 2021-09-06

**Authors:** Goutham Venkata Naga Davuluri, Chien-Chin Chen, Yen-Cheng Chiu, Hung-Wen Tsai, Hung-Chih Chiu, Yuh-Ling Chen, Pei-Jane Tsai, Wan-Ting Kuo, Nina Tsao, Yee-Shin Lin, Chih-Peng Chang

**Affiliations:** ^1^The Institute of Basic Medical Sciences, College of Medicine, National Cheng Kung University, Tainan, Taiwan; ^2^Department of Pathology, Ditmanson Medical Foundation Chia-Yi Christian Hospital, Chiayi City, Taiwan; ^3^Department of Cosmetic Science, Chia Nan University of Pharmacy and Science, Tainan, Taiwan; ^4^Division of Gastroenterology and Hepatology, Department of Internal Medicine, National Cheng Kung University Hospital, College of Medicine, National Cheng Kung University, Tainan, Taiwan; ^5^Department of Pathology, College of Medicine, National Cheng Kung University, Tainan, Taiwan; ^6^Institute of Oral Medicine, College of Medicine, National Cheng Kung University, Tainan, Taiwan; ^7^Department of Medical Laboratory Science and Biotechnology, National Cheng Kung University, Tainan, Taiwan; ^8^Department of Medical Laboratory Science, College of Medicine, I-Shou University, Kaohsiung, Taiwan; ^9^Department of Microbiology and Immunology, College of Medicine, National Cheng Kung University, Tainan, Taiwan

**Keywords:** galectin-1, hepatocellular carcinoma, secretory autophagy, tumor-associated macrophages, toll-like receptor 2

## Abstract

Galectin-1 (Gal-1) is a secretory lectin with pro-tumor activities and is associated strongly with hepatocellular carcinoma (HCC) development. Although Gal-1 is a well-known soluble pro-tumor factor in the tumor microenvironment (TME), the secretion mode of Gal-1 is not clearly defined. On the other hand, in addition to cancer cells, Gal-1 is widely expressed in tumor stromal cells, including tumor-associated macrophages (TAMs). TAMs are a significant component of stromal cells in TME; however, their contributions in producing Gal-1 to TME are still not explored. Here we reveal that TAMs can actively secrete Gal-1 in response to stimuli of HCC cells. Gal-1 produced by TAMs leads to an increase of the systemic level of Gal-1 and HCC tumor growth in mice. Mechanistically, TLR2-dependent secretory autophagy is found to be responsible for Gal-1 secretion from TAMs. Gal-1 acts as a cargo of autophagosomes to fuse with multivesicular bodies via Rab11 and VAMP7-mediated vesicle trafficking before being secreted. This autophagy-regulated Gal-1 secretion in TAMs correlates to poor overall survival and progression-free survival rates of HCC patients. Our findings uncover the secretion mode of Gal-1 via secretory autophagy and highlight the pathological role of TAM-produced Gal-1 in HCC progression.

## Introduction

Hepatocellular carcinoma (HCC) is one of the most common primary hepatic malignancies. The 5-year survival rate of patients with HCC in United States is less than 20% (Siegel et al., 2018). Prevalence and mortality of HCC have been increased due to many risk factors, including hepatitis viral infection, alcohol-related cirrhosis, and non-alcoholic fatty liver diseases (Sposito et al., 2019). Many of the patients suffering from HCC were diagnosed at intermediate and advanced stages of tumor; however, the effective treatments for these patients are limited because of its complex pathogenesis (Dhanasekaran et al., 2016). Of note, emerging evidence has defined the crucial role of the tumor microenvironment (TME) in the development and progression of HCC (Santhakumar et al., 2020). The cellular and non-cellular components of the tumor niche supporting the tumor growth and metastasis comprise TME, where dynamic interactions between cancer cells and immune cells significantly impact tumor growth and therapy efficacy (Wang et al., 2017; Arora and Pal, 2021). A significant subpopulation of immune cells in TME is that of tumor-associated macrophages (TAMs), whose function is evidently to promote the tumor progression, even though it was first thought to be a host immune response against the growing tumors. Accordingly, these TAMs are associated with poor prognosis of HCC patients, which makes them potential therapeutic targets to abrogate the development of cancer (Zhang et al., 2019; Allavena et al., 2021; Huang et al., 2021). TAMs have been considered to exhibit the pro-tumor functions in supporting tumor growth and metastasis and suppressing anti-tumor immunity. Accumulated studies have shown that upregulation of programmed cell death 1 ligand 1 (PD-L1), Toll-like receptors (TLRs), and T cell immunoglobulin mucin-3 (Tim-3) on TAMs affects antigen presentation, accumulation of Tregs cells, and facilitating M2 macrophage polarization (Yan et al., 2015; Doherty, 2016; Chew et al., 2017). In addition, TAMs can secrete various immunosuppressive mediators, such as interleukin-10 (IL-10), transforming growth factor-beta (TGF-β), and prostaglandin E2 (PGE2), into the TME to further inhibit anti-tumor immunity (Zhou et al., 2021).

Galectins are a physiologically conserved family of lectins sharing consensus amino acid sequences, including carbohydrate recognition domain (CRD), responsible for β-galactoside binding. Fifteen mammalian galectins have been identified to date, comprising monomers, homodimers, and oligomers. By binding to glycosylated proteins and lipids, these galectins are critical players in regulating various biological reactions (Compagno et al., 2020). Although galectins lack the signal sequence for secretion, some galectins are found in the extracellular space, including galectin-1 (Gal-1) (Hughes, 1999). Gal-1, the first discovered member of the galectin family, is widely distributed in the immune system and other tissues to contribute to its crucial function (Camby et al., 2006). For example, Gal-1 can facilitate macrophage reprogramming and provides a powerful anti-inflammatory effect by suppressing T cells (Camby et al., 2006; Yaseen et al., 2020). Notably, overexpression of Gal-1 is linked to the promotion of many types of cancer progression. Gal-1 in TME has been revealed to support tumor growth by increasing cell adhesion, invasiveness, angiogenesis, and evasion of immune surveillance (Goud et al., 2019; Martinez-Bosch and Navarro, 2020). Recent reports further explored that Gal-1 is essential for inducing the sorafenib and cisplatin resistance in HCC (Su et al., 2016; Zhang et al., 2016). However, the process that mediates the secretion of Gal-1 in TME is still not well-defined.

Given that Gal-1 lacks a recognizable signal sequence to pass through the endoplasmic reticulum-Golgi route, the export of this protein was suggested to undergo an unconventional secretory pathway (Popa et al., 2018). Accordingly, it has been recently revealed that a dectin-1-dependent galectin-3 secretion is inhibited in autophagy-deficient macrophages, which implies involvement by secretory autophagy in galectin secretion (Ohman et al., 2014). Autophagy is a well-regulated cellular system that was once viewed exclusively for its role in the cytoplasmic auto-digestive process to maintain the homeostasis of a cell. The autophagy process is controlled by a family of autophagy-related proteins (ATG) to mediate the package of cargo proteins in a double membraned autophagosome and their subsequent degradation by fusion with lysosomes. However, instead of being degraded, some cargo proteins can be secreted out by autophagy, in a process termed secretory autophagy (Kawabata and Yoshimori, 2020; Padmanabhan and Manjithaya, 2020). One of the earliest examples of autophagy-dependent unconventional secretion described in mammalian cells is IL-1β which lacks a leader peptide and is processed through a cytoplasmic platform termed inflammasome (Zhang et al., 2015). The biogenesis of autophagosomes by ATG protein families, such as ULK, Beclin-1, ATG5, ATG16 LC3, and GABARAP, is essential in this unconventional secretory pathway. The cargo-carrying autophagosomes are fused with the multivesicular bodies (MVBs) to form amphisome before secretion. Several vesicular trafficking regulators, including small GTPase Rab protein family and SNARE proteins, have been revealed to regulate secretory or degradative autophagy. For example, Rab11 participates in amphisome formation, and Rab8a mediates transporting amphisome to the plasma membrane, while syntaxin 17 (Stx17) and Rab8b are responsible for fusion of lysosome and autophagosome (Amaya et al., 2015; Padmanabhan and Manjithaya, 2020). It is still unclear whether secretory autophagy is involved in Gal-1 secretion.

Here, we examined the contribution of TAM-produced Gal-1 in HCC progression and the secretion mode of Gal-1. We reported that HCC cells stimulate TAMs to actively secrete Gal-1 via TLR2-triggered secretory autophagy and MVB/Rab11/VAMP7-mediated vesicle trafficking. The TAM-secreted Gal-1 by autophagy promotes HCC growth in mice and correlates to poor prognosis of HCC patients. Our findings explore the secretion mode of Gal-1 secretion and its pathological role in TAM-associated HCC progression.

## Materials and Methods

### Reagents and Antibodies

Brefeldin A, 3-methyladenine (3-MA), MG132, N-acetyl-l-cysteine, puromycin, and DAPI stain solution were purchased from Sigma-Aldrich (St. Louis, MO, United States). 2′,7′-dichlorodihydrofluorescein diacetate (H2DCFDA) and Lipofectamine^®^ 3000 transfection reagents were purchased from Thermo Fisher Scientific Inc., (Waltham, MA, United States). Small interfering RNA of Stx17 and scramble were purchased from Thermo Fisher Scientific Inc., (Waltham, MA, United States). Small interfering RNA of CD63 and Rab11 were purchased from Santa Cruz Biotechnology, Inc., (Dallas, TX, United States). Recombinant mouse macrophage colony-stimulating factor (M-CSF) was purchased from PeproTech (Cranbury, NJ, United States). Antibodies against Gal-1, Stx17, CD63, Rab11, VAMP7, calreticulin, heat shock protein 60 (HSP60), and β-actin were purchased from Abcam (Cambridge, United Kingdom). Gold nanoparticles (20 nm) conjugated secondary antibodies were also purchased from Abcam (Cambridge, United Kingdom). Antibodies against LC3, Atg5, and p62 were purchased from MBL (Nagoya, Japan). Antibodies against SOCS3 were purchased from the Proteintech group (Rosemont, IL, United States). Antibodies against CD68 were purchased from Agilent (Santa Clara, CA, United States). Mouse IL-10 was detected by ELISA (R&D Systems, Minneapolis, MN, United States).

### Cell Culture

Mouse hepatoma cell line ML-1_4a_ was adapted from ML-1 cells in BALB/c mice (Chang et al., 2013). Mouse macrophage cell line RAW 264.7 and human embryonic kidney cells 293T were purchased from the American Type Culture Collection Cell bank. Wild type and Atg5-/- mouse embryonic fibroblasts (MEF) were kindly provided by Dr. Tamotsu Yoshimori (Osaka University, Japan). MEF, ML-1_4a_, and 293T cells were cultured in Dulbecco’s modified Eagle medium (DMEM) while RAW 264.7 cells were cultured in RPMI 1640 medium supplemented with 10% heat-inactivated fetal bovine serum (FBS), 0.05 mg/ml streptomycin, and 50 U/mL penicillin. To generate mouse bone marrow-derived macrophages (BMDMs), bone marrow cells were collected from femurs and tibias of 6- to 10-week-old mice. These cells were cultured for 5 to 6 days in RPMI 1640 medium supplemented with 10% heat-inactivated FBS and 10 ng/ml recombinant mouse M-CSF to differentiate into BMDMs.

### Animal Studies

C57BL/6 mice and athymic nude mice (8–10 weeks old) were purchased from the National Laboratory Animal Center, Taiwan. Gal-1 knockout mice with C57BL/6 background were obtained from the Mutant Mouse Regional Resource Center and maintained in the Animal Laboratory of National Cheng Kung University. TLR2 knockout mice with C57BL/6 background were kindly provided by Dr. John T. Kung (Institute of Molecular Biology, Academia Sinica, Taiwan). TLR4 knockout mice with C57BL/6 background were kindly provided by Dr. Akira Shizuo (Laboratory of Host Defense, WPI Immunology Frontier Research Center, Osaka University). All mice were raised and cared for according to the guidelines set up by the Institutional Animal Care and Use Committee (IACUC) of National Cheng Kung University, and the Committee also approved this study (Ethics of Animal Experiments of National Cheng Kung University, Permit Number:107130). To examine the Gal-1 role in TAMs, 5 × 10^5^ ML-1_4a_ cells were mixed with 5 × 10^5^ wild type or Gal-1-/- BMDMs and then subcutaneously injected into nude mice. The tumor volume in HCC-bearing nude mice was monitored.

### Lentivirus-Based Short Hairpin RNA and Sirna Transfection

Atg5 and VAMP7 were knocked down in RAW 267.4 cells by stably expressing lentivirus-based shRNA. The clones were obtained from the National RNAi Core Facility (Institute of Molecular Biology/Genomic Research Center, Academia Sinica, Taiwan) and the target sequence for Atg5 was TRCN 0000375819 5′-AGCCGAAGCCTTTGCTCAATG -3′, while for VAMP7 it was TRCN 0000353419 5′-GCACTTCCTTATGCTATGAAT-3′, and for control luciferase it was TRCN0000072247 5′-GAATCGTCGTATGCAGTGAAA-3′. To generate shRNA carrying lentivirus, 293T cells (1 × 10^6^) were seeded into a 6 cm-plate, and then DNA mixture in 10 μL GeneJammer transfection reagent (Agilent, CA, United States) was added (pCMVdeltaR8.91: 0.9 μg/well; pMD.G: 0.1 μg/well; shRNA: 1 μg/well). After 48 h of incubation, the supernatants were collected to concentrate the virus. RAW 267.4 cells were infected with the lentivirus in the presence of polybrene (8 μg/ml) at 37°C for another 48 h. The cells were cultured with puromycin (5 μg/ml) containing medium to select stably expressing lentivirus-based shRNA. Stx17, CD63, and Rab11 were silenced in RAW 267.4 cells by transfecting small interfering RNA. The siRNA was packaged in liposomes by Lipofectamine^®^ 3000 transfection reagent according to the manufacturer’s instructions. The silencing efficacy of these proteins was determined by Western blotting.

### Autophagosome Isolation

Raw 264.7 cells (2 × 10^6^/per 10 cm dish) were treated with ML-1_4a_ hepatoma culture medium (MCM) for 6 h. The cells were suspended in 10% sucrose and mixed with 0.5 ml of the buffer (1 M Hepes/0.1 M EDTA), and then cells were physically broken down by using a 25G syringe. This homogenate was diluted with homogenization buffer (0.25 M sucrose, 10 mM Hepes, 1 mM EDTA, pH = 7.3) containing 0.5 mM glycyl-l-phenylalanine 2-naphthylamide (Cayman Chemical, MI, United States) and 1% dimethyl sulfoxide. After incubation for 7 min at 37°C, this homogenate was centrifuged at 4000 rpm for 2 min to collect the supernatant (post-nuclear supernatant). The supernatant was added into a tube with 9.5% Nycodenz (Alere Technologies AS, Norway) (middle layer) and 22.5% Nycodenz (bottom layer). The Nycodenz gradient was centrifuged at 28000 rpm in an SW28 rotor overnight, and then three fractions were observed. The interface band was added to the tube with 33% Percoll (middle layer) and 22.5% Nycodenz (bottom layer) and then centrifuged for 1 h at 20000 rpm in the SW28 rotor to collect the autophagosome-enriched layer.

### Western Blotting

The cells were collected for homogenization in a cell lysis buffer (Cell Signaling Technology, MA, United States) on ice for 20 min, and then centrifuged at 13000 rpm under 4°C for 20 min to collect the supernatants. Proteins were separated through SDS gel electrophoresis and transferred onto the PVDF membranes. The PVDF membranes were blocked with 5% skimmed milk and incubated with the appropriate primary antibodies at 4°C overnight. The membranes were then washed and incubated with peroxidase-conjugated secondary antibodies. The blots were visualized by enhancing chemiluminescence reagents (PerkinElmer Life Sciences, Hopkinton, MA, United States).

### Immunofluorescence Stain

To monitor the protein distribution in macrophages, BMDMs or RAW264.7 cells were seeded into a cover slide, fixed with 4% formaldehyde, and stained with primary antibodies against Gal-1, LC3, CD63, Rab11, and VAMP7 at 4°C overnight. For detection of the expression of Gal-1 and LC3 in TAMs of human HCC, paraffin-embedded HCC tissue sections were deparaffinized with xylene and alcohol, and then antigen retrieval was performed with 10 mM citrate buffer at 95–100°C for 10 min. Next, the tissue sections were stained with primary antibodies against Gal-1, LC3, and CD68 at 4°C overnight. All above slides were stained with fluorescent dye-conjugated secondary antibodies at room temperature for 2 h. The protein distribution was determined by a confocal fluorescence microscope (Olympus FV 3000, Japan). The percentages of LC3-positive or Gal-1-positive cells in CD68-positive TAMs were visually evaluated and digitally documented with merged images. Further, we defined 50% as the cut-off point, e.g., the high Gal-1 expression (>50%) and the low Ga1-1 expression (≦50%). Subsequently, we divided the 93 HCCs into four groups, (1) low Gal-1 and low LC3 (Gal-1^–^ LC3^–^), (2) low Gal-1 and high LC3 (Gal-1^–^ LC3^+^), (3) high Gal-1 and low LC3 (Gal-1^+^ LC3^–^), and (4) high Gal-1 and high LC3 (Gal-1^+^ LC3^+^).

### Immunoelectron Microscopy

For immunoelectron microscopy analysis, macrophages were fixed in 4% formaldehyde and 1% glutaraldehyde in 0.1 M PBS, followed by dehydration and sectioning. The cell sections were first blocked with 1% BSA at room temperature for 1 h. Next, the primary antibodies against Gal-1 were co-incubated with sections at 4°C overnight. After washing steps, the sections were incubated with gold nanoparticles (20 nM) conjugated secondary antibodies at room temperature for 2 h. Sections were further stained with uranyl acetate for 20 min and lead citrate for 4 min and then examined using a JEM-1400 transmission electron microscope (Japan Electron Optics Laboratory Co., Ltd., Japan).

### Patient Samples and Clinical Information

The institutional review board approved the study design and patient recruitment (CYCH-IRB No. 106-030 and CYCH-IRB No. 104079, Ditmanson Medical Foundation Chia-Yi Christian Hospital, Taiwan). Twenty-one healthy volunteers and 25 HCC patients were recruited to compare the serum level of Gal-1. A total of 93 surgically treated patients with HCCs were retrospectively enrolled at Ditmanson Medical Foundation Chia-Yi Christian Hospital, Taiwan, from January 2007 to December 2012. The inclusion criteria included: (1) a tissue-proved HCC after liver resection; (2) medical histories and therapeutic outcomes were complete and well traceable; (3) at least 1 month of post-operation survival; and (4) no neoadjuvant therapies before surgery. The exclusion criteria included: (1) metachronous or synchronous tumor in other organs; (2) a mixed type of liver tumor. Clinical data, including gender, age, viral status (hepatitis B virus and hepatitis C virus), tumor size, stage, survival time, disease recurrence and progression, image features, and treatment response, were obtained by chart review. The tumor stage was re-evaluated according to the eighth edition of the American Joint Commission on Cancer (AJCC) staging system. Histopathological characteristics, including diagnosis, the modified histology activity index (HAI) score, and the presence of cirrhosis, were re-evaluated by an experienced pathologist blinded to the clinical information. The clinical and pathologic parameters of the patients are shown in Supplementary Table 1. All patients were followed till the death or till February 2017 and the follow-up time was between 1–120 months (median, 68 months).

### Gene Set Enrichment Analysis

Gene Set Enrichment Analysis (GSEA) was performed on RNA-Seq gene expression data from The Cancer Genome Atlas (TCGA) liver cancer dataset. GSEA was performed to determine galectin-1 enrichment in gene lists extracted from MSigDB (Mootha et al., 2003; Subramanian et al., 2005) in order to determine the enrichment in gene sets from the immunologic signatures (C7) collection.

### Statistical Analysis

All cell and mouse experiments were performed at least two to three times, and the data are expressed as means ± SD. Data were analyzed by One-Way ANOVA using GraphPad Prism 8 software (GraphPad Software Inc., San Diego, CA, United States), and *p* < 0.05 was considered to represent significant differences between groups. Regarding the clinicopathological characteristics, data were analyzed using one-way ANOVA for continuous variables and the Fisher exact test for categorical variables to evaluate the association between different groups with different Gal-1/LC3 expression levels. In addition, Spearman rank-order correlation analysis was employed to explore the relationship between Gal-1 and LC3 in TAMs. Progression-free survival (PFS) was the interval between tumor resection and the first time of either disease progression recurrence, relapse, or death from any cause. Overall survival (OS) was defined as the time from surgery to the date of death. The follow-up time was defined as the period between surgery and the end of follow-up or death. The OS and PFS rates were calculated using the Kaplan–Meier method with Bonferroni adjustment for multiple pairwise comparisons. Hazard ratios (HRs) and confidence intervals (CIs) were calculated using the Cox regression method. Statistical analyses were performed using SPSS (version 22.0; IBM Inc.) or GraphPad Prism 8 software (GraphPad Software Inc., San Diego, CA, United States), and two-tailed *p* < 0.05 was considered to represent significant differences between groups.

## Results

### Hepatoma Cells Stimulate Macrophages to Actively Secrete Gal-1 to Promote Tumor Growth

**FIGURE 1:**
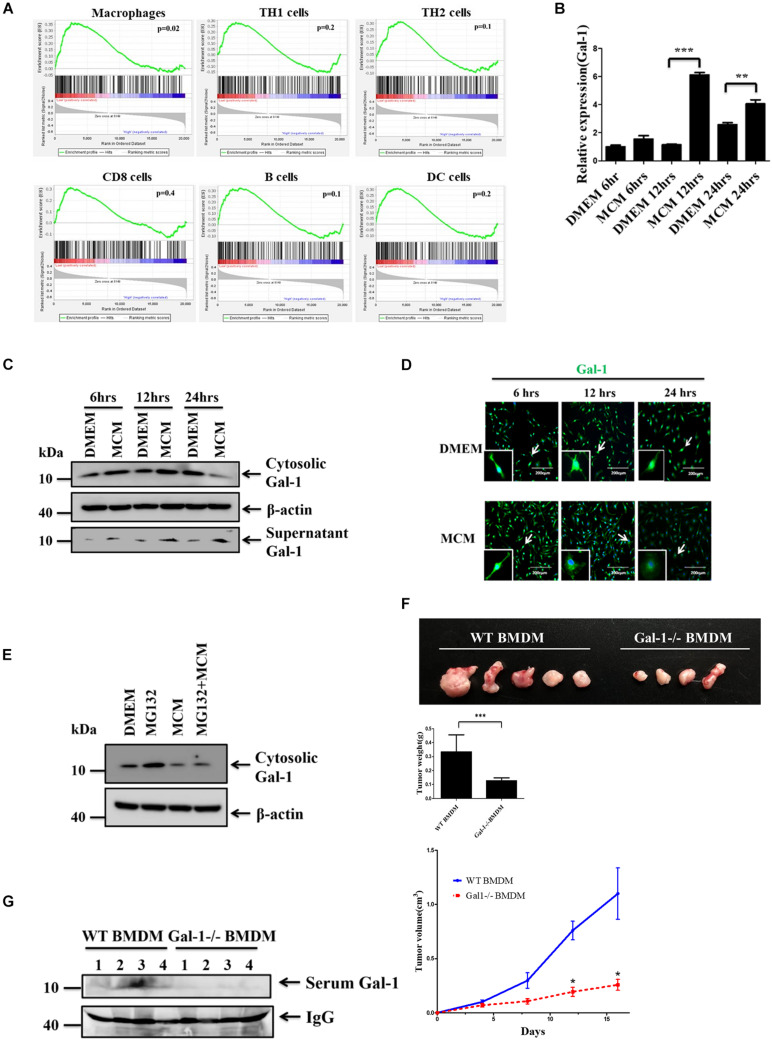
HCC cells stimulate macrophages to actively secrete Gal-1. **(A)** GSEA of Gal-1 mRNA expression in HCC-associated immune cells. The data of Gal-1 mRNA expression in human liver cancer patients was obtained from the cancer genome atlas (TCGA) database. Enrichment scores (ES) are shown on the y-axis. X-axis bars represent individual genes of immunologic signature gene sets. Color bars represent the expression level of Gal-1. Positive and negative ES indicate enrichment of immunologic signature gene sets in high expression galectin-1 and low expression galectin-1, respectively. **(B–D)** BMDMs were treated with MCM at indicated time. The expression levels of Gal-1 mRNA were determined by qRT-PCR and quantified **(B)**. The expression of cytosolic Gal-1, β-actin as well as supernatant Gal-1 proteins was determined by Western blotting **(C)** or immunofluorescence staining **(D)**. All blots were derived from a single experiment. **(E)** BMDMs were pretreated with or without MG132 (0.25 μM) for 1 h prior to adding MCM. The expression of cytosolic Gal-1 and β-actin was determined by Western blotting. All blots were derived from a single experiment. **(F,G)** 5 × 10^5^ ML-1_4a_ cells mixed with 5 × 10^5^ WT or Gal-1 -/- BMDMs were subcutaneously inoculated into nude mice (*n* = 5). Tumor volume was measured every 4 days after inoculation. All mice were sacrificed on day 16. The representative images of HCC tumors were shown, and the tumor weights were quantified **(F)**. The expression of galectin-1 in mice serum was measured by Western blotting **(G)**. All blots were derived from a single experiment. *p < 0.01; **p < 0.001; ***p < 0.0001.

Upregulated expression of Gal-1 is found in the tumor area and peripheral blood of HCC patients, and this correlates with the poor prognosis of these patients (Wu et al., 2012). Immune cells are known to express and produce Gal-1 in response to stimuli (Thiemann and Baum, 2016), but their roles in upregulated Gal-1 observed in HCC are still a puzzle. To assess Gal-1 expression in HCC-associated immune cells, we analyzed the relationship of Gal-1 mRNA expression to various immune subpopulation markers of human HCC from available RNAseq data in TCGA by GSEA analysis (Figure 1A). We found a high correlation of Gal-1 expression to macrophage-rich HCC tumors, as identified by gene signatures, but not to Th1, Th2, CD8^+^ T, B cells, or dendritic cells (Figure 1A). This indicates that HCC-associated macrophages may contribute to the increase of Gal-1 in TME. To investigate this process, we have introduced a co-culture system in which the bone marrow-derived macrophages (BMDMs) were co-cultured with MCM to differentiate as the M2-like phenotype with increased expression of SOCS3 and IL-10 (Supplementary Figures 1A,B) as we previously reported (Chang et al., 2013). The expression and secretion levels of Gal-1 in MCM-treated BMDMs were then monitored. As the results showed in Figure 1B, MCM treatment stimulated up-regulation of Gal-1 mRNA in BMDMs, which is consistent with findings in Figure 1A. By Western blot analysis, the protein level of Gal-1 was increased at 6 and 12 h in MCM-treated BMDMs; however, the level was gradually reduced at 24 h (Figure 1C). A similar dynamic expression pattern of Gal-1 was observed by immuno-fluorescent analysis (Figure 1D). Since Gal-1 mRNA was not down-regulated at 24 h in MCM-treated BMDMs (Figure 1B), we examined whether the reduction of the protein level of Gal-1 was caused by protein instability. We found no restoration of Gal-1 in MCM-treated macrophages in the presence of proteasome inhibitor MG132 (Figure 1E). These findings suggested that cytosolic Gal-1 may be secreted from MCM-treated macrophages. As expected, a significant increase of supernatant Gal-1 was observed in MCM-treated BMDMs and RAW264.7 cells (Figure 1C and Supplementary Figures 2A,B). To rule out the possibility of passive release of Gal-1, we checked cell death by LDH release analysis in which MCM was not able to induce cell death (Supplementary Figure 2C). All these data collectively indicate that HCC cells are capable of triggering macrophages to secrete Gal-1 actively. Next, we further examined whether Gal-1 produced by HCC-associated macrophages contributes to HCC progression. Consequently, the ML-1_4a_ hepatoma cells were mixed with wild type (WT) or Gal-1-/- BMDMs and then inoculated subcutaneously into nude mice. As indicated by the results shown in Figures 1F,G, mice carrying ML-1_4a_ tumors co-injected with Gal-1-/- BMDMs displayed reduced tumor growth, size, and weight compared with co-injection with WT BMDMs. Furthermore, the serum level of Gal-1 was significantly decreased in HCC-bearing mice co-injected with Gal-1-/- BMDMs compared with co-injection of WT BMDMs (Figure 1H). These results indicate that Gal-1 produced by HCC-associated macrophages facilitates HCC progression.

### Autophagy Regulates the Secretion of Gal-1 From Hepatoma Cell-Stimulated Macrophages

**FIGURE 2:**
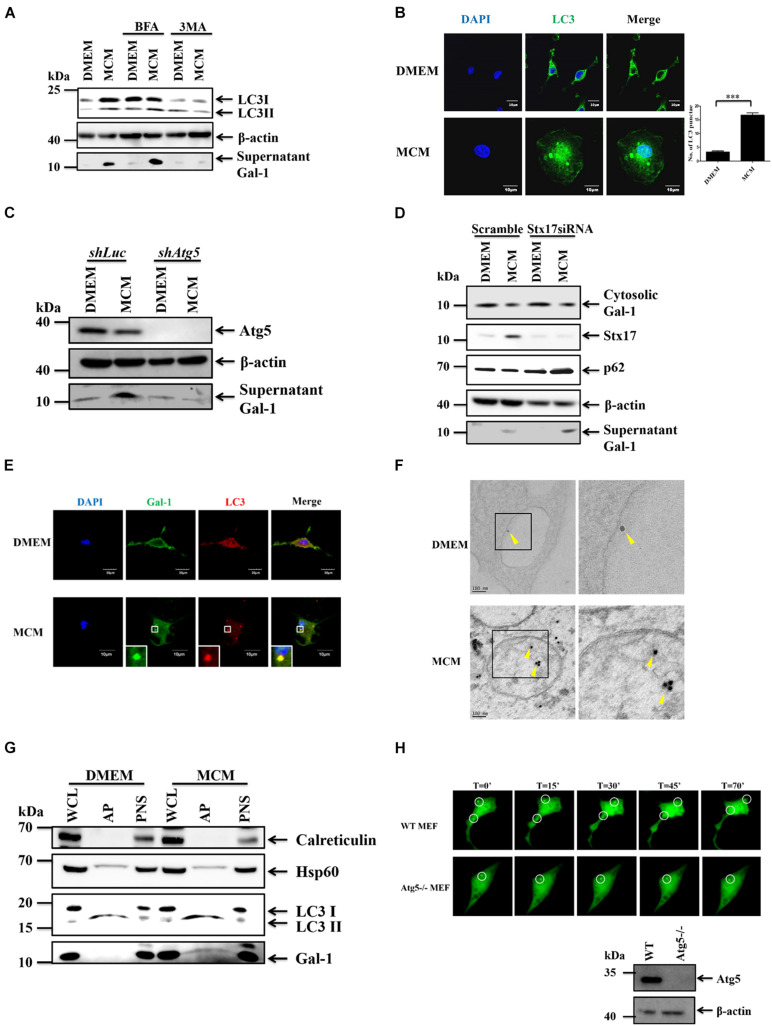
Gal-1 secretion is mediated by autophagy. **(A)** BMDMs were pretreated with 3MA (2 mM) or Brefeldin A (30 nM) for 1 h and then treated with MCM for another 24 h. The expression of cytosolic LC3-I/II, β-actin, and supernatant G-1 proteins was determined by Western blotting. All blots were derived from a single experiment. **(B)** BMDMs were treated with MCM for 12 h and stained with anti-LC3 antibody. The LC3 aggregation (green) was observed by confocal microscopy. Scale bar = 10 μm. **(C)** RAW 264.7 cells stably expressing luciferase (Luc) or Atg5 shRNA were treated with MCM for 24 h. The expression of cytosolic Atg5, β-actin, and supernatant Gal-1 proteins was determined by Western blotting. All blots were derived from a single experiment. **(D)** RAW264.7 cells were transfected with control or Stx17 siRNA at 10 μM for 24 h and then treated with MCM for another 24 h. The expression of cytosolic Gal-1, Stx17, p62, β-actin, and supernatant Gal-1 proteins was determined by Western blotting. All blots were derived from a single experiment. **(E)** BMDMs were treated with MCM for 12 h and stained with anti-Gal-1 (green) and -LC3 (red) antibodies. The distribution of these proteins was analyzed by confocal microscopy. Scale bar = 10 μm. **(F)** BMDMs were treated with MCM for 6 h to detect Gal-1 by immunoelectron microscopy. Gold nanoparticle-labeled Gal-1 (arrow bead) in the autophagosomes was observed under a transmission electron microscope. Scale bar = 60 nm. **(G)** RAW 264.7 cells were treated with MCM for 6 h to isolate autophagosomes. The expression of calreticulin, HSP60, LC3-I/II, and Gal-1 proteins in different cell components was determined by Western blotting. WCL: whole cell lysates; PNS: post-nuclear supernatants; AP: autophagosomes. All blots were derived from a single experiment. **(H)** WT and Atg5-/- MEF cells were transfected with Gal-1-GFP to monitor the Gal-1 trafficking by TIRF microscopy. Selected frames from time-lapse TIRF microscopy with time intervals in seconds are shown. The protein expression of Atg5 of WT and Atg5-/- MEFs was determined by Western blotting (bottom right corner). All blots were derived from a single experiment.

It is known that Gal-1 lacks a specific signal sequence for secretion via the conventional pathway, which suggests the involvement of unconventional secretion pathways, such as secretory autophagy (Martinez-Bosch and Navarro, 2020). To determine the secretory pathway of Gal-1 in MCM-treated macrophages, the autophagy inhibitor, 3-MA, and the classical secretion pathway inhibitor, brefeldin A (BFA), were used. Our data revealed that treatment of 3-MA, but not BFA, significantly inhibited the secretion of Gal-1 in MCM-treated macrophages (Figure 2A). Accordingly, the upregulated expression of LC3 puncta in MCM-treated macrophages was observed by confocal microscopy (Figure 2B). Consistent with the 3-MA results described above, silencing of Atg5 in macrophages reduced MCM-induced Gal-1 secretion as well (Figure 2C). These data indicate that autophagy plays a role in regulating this Gal-1 secretion. To understand whether this autophagy-regulated Gal-1 secretion relies on the process of lysosome fusion, we knocked down syntaxin 17 (Stx17), a SNARE protein that is essential to mediate fusion of lysosomes with autophagosomes (Itakura et al., 2012), by transfection of siRNA targeting Stx17. Knockdown of Stx17 resulted in an increase of accumulation of p62 in macrophages, indicating the blockage of fusion between lysosomes and autophagosomes. However, these Stx17-knockdown macrophages did not suppress Gal-1 secretion after MCM treatment compared with scramble siRNA transfecting control cells, indicating that autolysosomes are not involved in the secretion of Gal-1 (Figure 2D). Next, we asked whether Gal-1 is a cargo protein of autophagosomes. Using immunostaining analysis, we found that Gal-1 was co-localized with LC3, and located inside the double-layer autophagosomes in MCM-treated macrophages, according to confocal microscopy and immuno-electron microscopy observations, respectively, (Figures 2E,F). In addition, the presence of Gal-1 in isolated autophagosomes of MCM-treated macrophages was increased compared to control cells (Figure 2G). For real-time visualization of the effect of autophagy on Gal-1 trafficking, we performed total internal reflection fluorescence (TIRF) microscopy analysis to selectively visualize labeled vesicles which are located close to the plasma membrane. To determine this, we transfected GFP-Gal-1 into WT or Atg5-/- mouse embryonic fibroblasts (MEFs) and tracked the movement of GFP-Gal-1 by TIRF microscopy. The expression of Atg5 in WT or Atg5-/- MEF cells was confirmed by immunoblots (Figure 2H). The GFP-Gal-1 dots moved quickly and diffused laterally in the TIRF evanescence field of WT MEF cells (Upper, Figure 2H and Supplementary Video 1), while the signal remains steady in the Atg5-/- cells (Lower, Figure 2H and Supplementary Video 2). These data apparently indicate that Gal-1 is carried by autophagosomes and is then subsequently released from HCC-stimulated macrophages.

### An MVB/ Rab11/VAMP7 Axis Controls the Secretion of Gal-1

**FIGURE 3:**
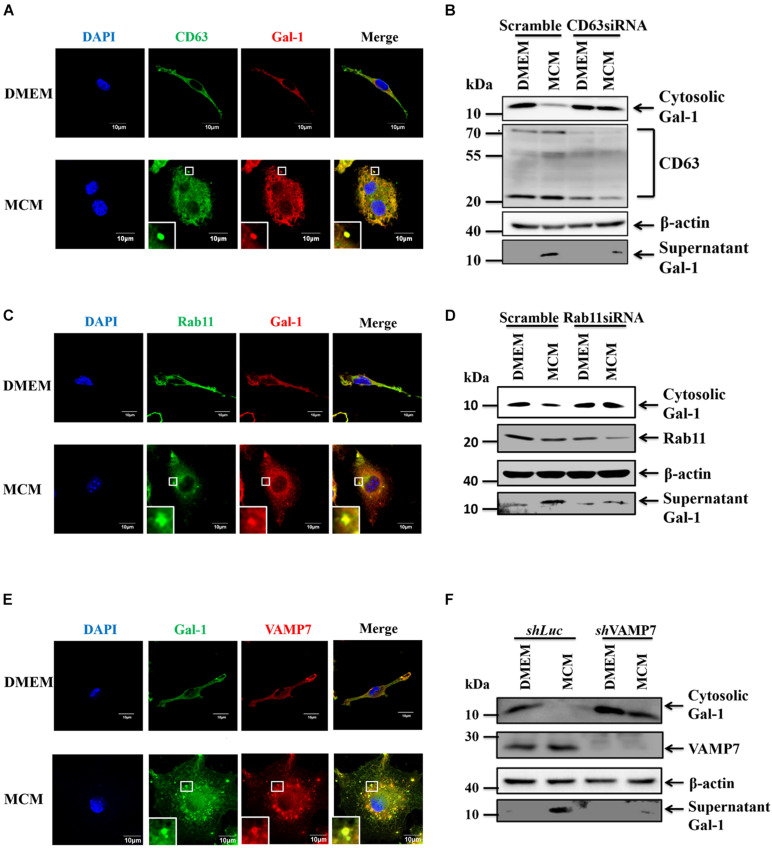
MVB, Rab11, VAMP7 triggered amphisome formation mediates Gal-1 secretion. **(A)** BMDMs were treated with MCM for 12 h and then stained with anti-CD63 (green) and anti-Gal-1 (red) antibodies. The co-localization of CD63 and Gal-1 was observed by confocal microscopy. Scale bar = 10 μm. **(B)** RAW 264.7 cells were transfected with control or CD63 siRNA for 24 h and then treated with MCM for another 24 h. The expression of cytosolic Gal-1, CD63, β-actin, and supernatant Gal-1 proteins was determined by Western blotting. All blots were derived from a single experiment. **(C)** BMDMs were treated with MCM for 12 h and then stained with anti-Rab11 (green) and anti-Gal-1 (red) antibodies. The co-localization of Rab11 and Gal-1 was observed by confocal microscopy. Scale bar = 10 μm. **(D)** RAW 264.7 cells were transfected with control or Rab11 siRNA for 24 h and then treated with MCM for another 24 h. The expression of cytosolic Gal-1, Rab11, β-actin, and supernatant Gal-1 proteins was determined by Western blotting. All blots were derived from a single experiment. **(E)** BMDMs were treated with MCM for 12 h and then stained with anti-Gal-1 (green) and anti-VAMP7 (red) antibodies. The co-localization of VAMP7 and Gal-1 was observed by confocal microscopy. Scale bar = 10 μm. **(F)** shLuc and shVAMP7 RAW264.7 cells were treated with MCM for 24 h. The expression of cytosolic Gal-1, VAMP7, β-actin, and supernatant Gal-1 proteins was determined by Western blotting. All blots were derived from a single experiment.

We next went further to address the pathways involved in autophagy-dependent Gal-1 secretion. MVBs are the endosome molecules that can interact with the autophagosomes to form hybrid structures called amphisomes to facilitate protein secretion (Hessvik and Llorente, 2018). Hence, we investigated whether the MVBs are involved in the secretion of Gal-1. To examine this, we first checked the co-localization of CD63, an MVB marker, and Gal-1 by confocal immunofluorescence microscopy. As shown by the results in Figure 3A, Gal-1 was found to co-localize with CD63 in MCM-treated macrophages. Then, the CD63 was knocked down by siRNA to examine MVB contribution on Gal-1 secretion. This was followed by Western blot analysis, where the recovery of cytosolic Gal-1 along with the reduction of supernatant Gal-1 was observed in CD63 silencing MCM-treated macrophages compared with control cells (Figure 3B). These data indicate the involvement of MVB in the secretion of Gal-1. Rab GTPase family proteins are well-studied key regulators of vesicle trafficking and protein secretion. One of these proteins, called Rab11, has been shown to play a pivotal role in amphisome-mediated protein secretion (Hessvik and Llorente, 2018). Thus, we examined whether Rab11 participates in Gal-1 secretion. According to our immunostaining observation, Rab11-positive vesicles were found to co-localize with Gal-1 (Figure 3C). Knockdown of Rab11 by siRNA significantly reduced secretion of Gal-1 from MCM-treated macrophages (Figure 3D), suggesting that Rab11 is essential for Gal-1 secretion. Furthermore, Rab11 is reported to interact with one of the SNARE proteins called VAMP7 to promote vesicle trafficking (Kandachar et al., 2018). Hence, we further investigated the role of VAMP7 in the secretion of Gal-1. Consistent with what we found in Figures 3C,D, the confocal image analysis has revealed a significant co-localization between the VAMP7 and Gal-1 (Figure 3E). Silencing of VAMP7 abrogated the supernatant level of Gal-1 in MCM-treated macrophages (Figure 3F). These results collectively suggest that an MVB/Rab11/VAMP7 axis facilitates the autophagy-mediated secretion of Gal-1.

### TLR2-Dependent Reactive Oxygen Species Regulate the Secretion of Gal-1

**FIGURE 4:**
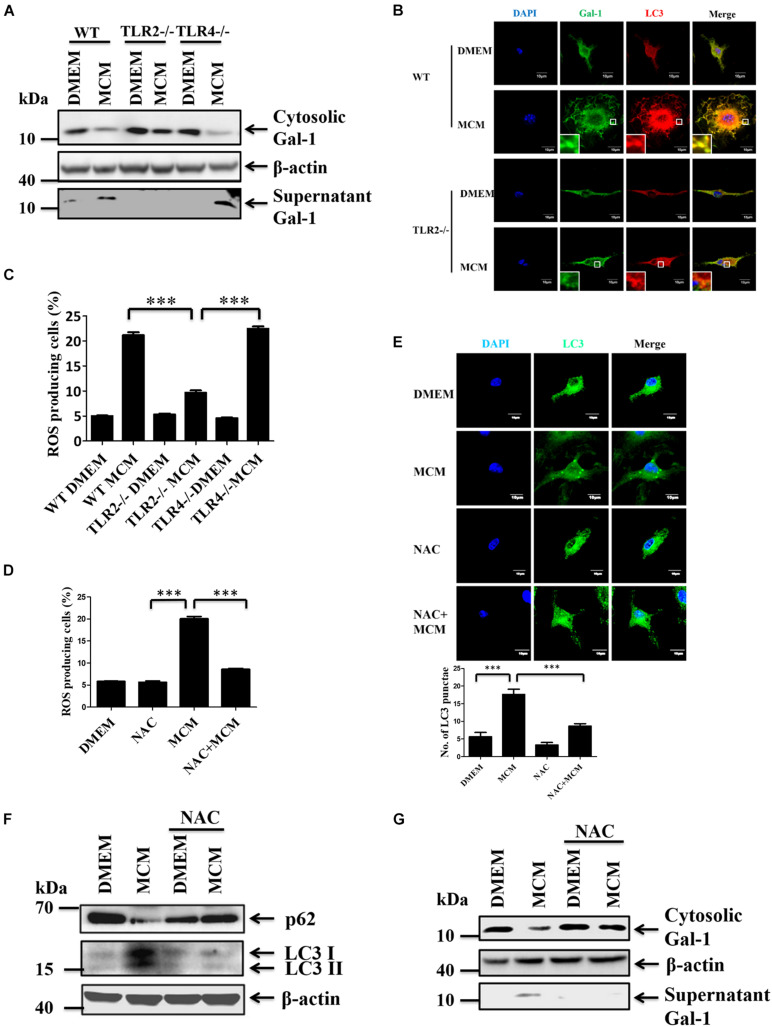
TLR2-mediated ROS production induces autophagy and Gal-1 secretion. **(A)** WT, TLR2-/- or TLR4-/- BMDMs were treated with MCM for 24 h. The expression of cytosolic Gal-1, β-actin, and supernatant Gal-1 proteins was determined by Western blotting. All blots were derived from a single experiment. **(B)** WT or TLR-/- BMDMs were treated with MCM for 12 h and then stained with anti-Gal-1 (green) and anti-LC3 (red) antibodies. The co-localization of Gal-1 and LC3 was observed by confocal microscopy. Scale bar = 10 μm. **(C)** WT, TLR2-/- or TLR4-/- BMDMs were treated with MCM for 12 h. The ROS production of these cells was analyzed by flow cytometry and quantified. **(D–F)** BMDMs were pretreated with or without NAC (4 mM) for 1 h and then treated with MCM for another 12 h. The ROS production of these cells was analyzed by flow cytometry and quantified **(D)**. The LC3 dots were observed by confocal microscopy and quantified **(E)**. The expression of cytosolic p62, LC3-I/II, and β-actin proteins was determined by Western blotting **(F)**. All blots were derived from a single experiment. **(G)** BMDMs were pretreated with or without NAC (4 mM) for 1 h and then treated with MCM for another 24 h. The expression of cytosolic Gal-1, β-actin, and supernatant Gal-1 proteins were determined by Western blotting. All blots were derived from a single experiment. ^∗∗∗^*p* < 0.0001.

We have recently demonstrated that TLR2 signal-induced reactive oxygen species (ROS) is essential to trigger autophagy in HCC-associated macrophages (Shiau et al., 2020). Thus, we examined whether TLR2 and ROS are responsible for mediating the autophagy-regulated secretion of Gal-1 from MCM-treated macrophages. Subsequently, the secretion of Gal-1 in MCM-treated wild type (WT), TLR2-/-, and TLR4-/- BMDMs was monitored. According to the results shown in Figure 4A, a significant decrease of Gal-1 secretion in MCM-treated TLR2-/- BMDMs was found compared with WT or TLR4-/- BMDMs. In addition, the LC3 puncta and co-localization of Gal-1 and LC3 were abolished in MCM-treated TLR2-/- BMDMs (Figure 4B). These data suggest that TLR2-dependent autophagy is crucial for Gal-1 secretion. To further address the role of ROS in Gal-1 secretion, we first monitored ROS production from MCM-treated WT, TLR2-/-, and TLR4-/- BMDMs. Our data showed that MCM triggers ROS generation in WT and TLR4-/- BMDMs, which was blocked in TLR2-/- macrophages (Figure 4C). Next, a well-known scavenger of ROS, N-acetyl cysteine (NAC), was used to inhibit MCM-triggered ROS in BMDMs (Figure 4D). Accordingly, the MCM-induced autophagy and Gal-1 secretion were determined. As shown by the results in Figures 4E,F, inhibition of ROS by NAC decreased the formation of LC3 puncta and LC3-II conversion in MCM-treated BMDMs compared with control cells. Furthermore, treatment of NAC was able to effectively suppress the Gal-1 secretion in MCM-treated BMDMs (Figure 4G). Together these findings suggest that TLR2-mediated ROS production is essential to induce autophagy-dependent secretion of Gal-1.

### Autophagy-Associated Gal-1 Expression in TAMs Is Linked to the Prognosis of HCC Patients

**FIGURE 5:**
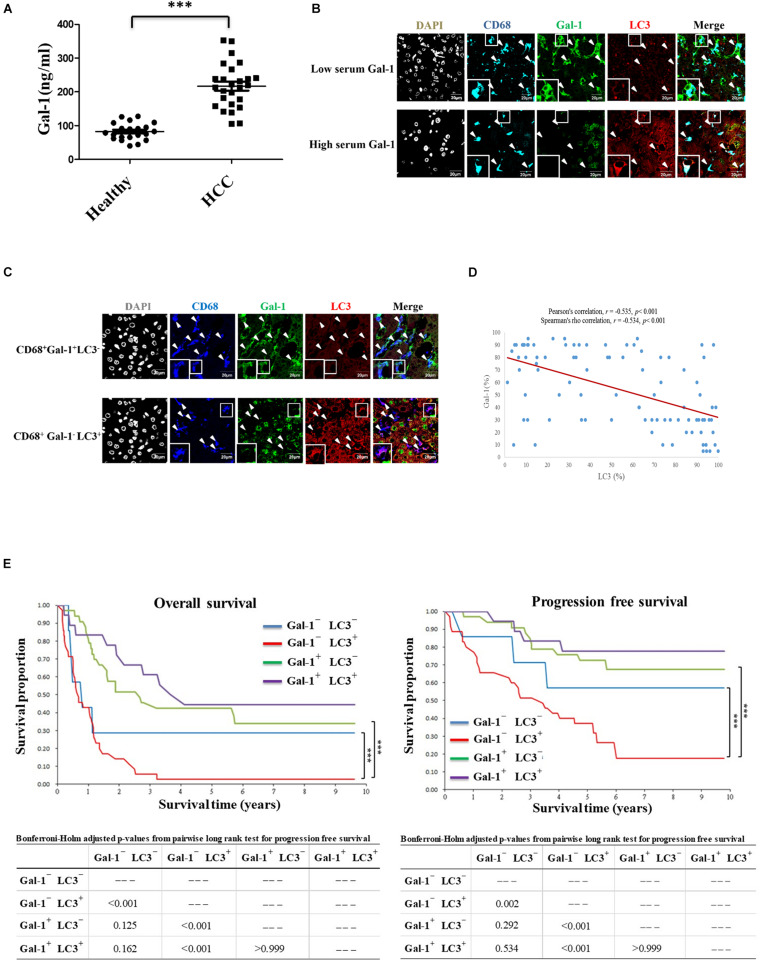
Intratumoral Gal-1^–^LC3^+^ TAMs correlate to the poor OS and PFS rates of HCC patients. **(A)** Serum levels of Gal-1 in healthy donors (*n* = 21) and HCC patients (*n* = 25) were analyzed by ELISA. ^∗∗∗^*p* < 0.0001. **(B)** Liver tumor sections from HCC patients with high or low serum levels of Gal-1 were stained with anti-CD68 (blue), anti-Gal-1 (green), and anti-LC3 (red) antibodies. Representative images show the protein distribution as observed by confocal microscopy. Scale bar = 20 μm. **(C–E)** Liver tumor sections of 93 retrospectively recruited HCC patients were stained with anti-CD68 (blue), anti-Gal-1 (green), and anti-LC3 (red) antibodies. Representative images show the protein distribution as observed by confocal microscopy (scale bar = 20 μm) **(C)**. The expression correlation between LC3 and Gal-1 in CD68^+^ TAMs was analyzed by Bivariate correlation analysis using Pearson’s correlation (r = −0.535, *p* < 0.001) as well as Spearman’s correlation (r = −0.534, *p* < 0.001) **(D)**. The OS and PFS rates of TAMs carrying Gal-1^–^ LC3^–^, Gal-1^–^ LC3^+^, Gal-1^+^ LC3^–^, or Gal-1^+^ LC3^+^ HCC patients were analyzed by Kaplan-Meier survival analyses **(E)**.

The relationship between autophagy and Gal-1 secretion has never been examined in TME of human HCC patients. Thus, in our pilot study, we first enrolled 25 HCC patients and 21 healthy volunteers to determine the serum level of Gal-1. In line with previous reports, an increased serum level of Gal-1 was found in HCC patients compared with healthy volunteers (Figure 5A). To examine the correlation between serum level of Gal-1 and autophagy activity in TAMs of HCC patients, we performed immunofluorescence staining in HCC patients’ tissues from those with high serum levels (over 250 ng/ml) or low serum levels (below 150 ng/ml) of Gal-1. Interestingly, we found a high proportion of Gal-1^–^LC3^+^CD68^+^ cells in HCC tissues with a high level of serum Gal-1 while Gal-1^+^LC3^–^CD68^+^ cells were rich in HCC tissues with a low level of serum Gal-1 (Figure 5B). The results suggest that autophagy activity in TAM may correlate to Gal-1 secretion in HCC patients. We next examined whether Gal-1^–^LC3^+^CD68^+^ cells correlate to HCC patients’ progression and poor prognosis. A total of 93 HCC patients were retrospectively recruited. The clinicopathological parameters of patients are summarized in Supplementary Table 1. Among 93 HCC patients, 66 (70.97%) were male, and 52 (55.91%) patients had liver cirrhosis with a mean age of 63.06 years. According to immunofluorescence staining of Gal-1 and LC3 on CD68^+^ TAMs, we divided the 93 HCCs into four groups, Gal-1^–^ LC3^–^, Gal-1^–^LC3^+^, Gal-1^+^ LC3^–^, and Gal-1^+^ LC3^+^. Although most clinicopathological variables of each group were not significantly different, there were differences in tumor size, stage, recurrence, and death between different groups. Groups with low Gal-1 expression (Gal-1^–^ LC3^–^ and Gal-1^–^ LC3^+^) had larger tumor size (*p* < 0.001), higher stage (*p* = 0.005), and higher frequencies of tumor recurrence (*p* = 0.033) and deaths (*p* < 0.001) than groups with high Gal-1 expression (Gal-1^+^ LC3^–^ and Gal-1^+^ LC3^+^) (Supplementary Table 1). Consistently, we found that high expressed Gal-1 TAMs (Gal-1^+^CD68^+^) showed a low level of LC3 while low expressed Gal-1 TAMs (Gal-1^–^CD68^+^) carried a high level of LC3 (Figure 5C). Notably, a significant negative correlation between Gal-1 and LC3 expression in TAMs of HCC patients was observed. Bivariate correlation analysis using Pearson’s correlation (r = −0.535, *p* < 0.001) implied a moderate negative correlation, yet a statistically significant linear relationship exists between LC3 and Gal-1. Moreover, Spearman’s correlation also demonstrated a statistically significant negative correlation (r = −0.534, *p* < 0.001) (Figure 5D). Moreover, Kaplan-Meier survival analyses revealed that the patients carrying Gal-1^–^ LC3^+^ TAMs had the worst overall survival (OS) and progression-free survival (PFS) rates, and the statistical significance was present in comparison with all three of the other groups (with the Gal-1^–^ LC3^–^ group: *p* < 0.001; with the Gal-1^+^ LC3^–^ group: *p* < 0.001; with Gal-1^+^ LC3^+^ group: *p* < 0.001) (Figure 5E). After adjusting for patients’ age, gender, tumor size, and stage, the Cox proportional Hazard model demonstrated that patients carrying Gal-1^–^LC3^+^ TAMs are significantly and independently at high-risk with a poor prognosis (Supplementary Table 2). Collectively, these data indicate that Gal-1^–^ LC3^+^ CD68^+^ cells serve as a prognostic factor for HCC’s poor progression.

## Discussion

**FIGURE 6:**
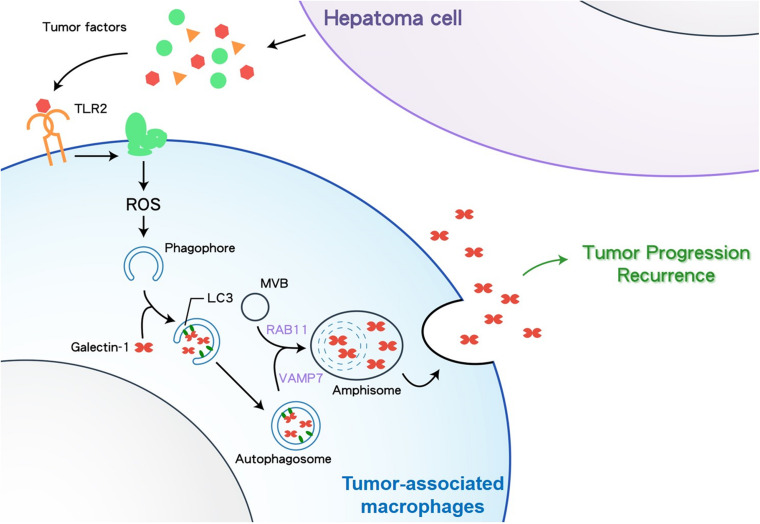
A schematic diagram depicting how HCC cells stimulate TAMs to secrete Gal-1 via secretory autophagy. TLR2, Toll-like receptor 2; ROS, reactive oxygen species; MVB, multivesicular body; VAMP7, Vesicle associated membrane protein 7.

Although Gal-1 is a well-known soluble pro-tumor factor in TME, the secretion mode of Gal-1 is not clearly defined. Here we reveal new findings that TAMs can secrete Gal-1 via secretory autophagy to facilitate HCC tumor growth. This autophagy-regulated Gal-1 secretion in TAMs requires TLR2-triggered ROS and MVB/Rab11/VAMP7-mediated vesicle trafficking. Clinically, compared to other phenotypes, HCC patients carrying Gal-1^–^ LC3^+^ CD68^+^ TAMs show the increased serum level of Gal-1 and worst OS and PFS rates (Figure 6). Our findings highlight the contribution of TAM-produced Gal-1 via secretory autophagy in HCC progression.

Galectin-1 has been found to accumulate in the stroma of HCC and is positively correlated with tumor size, stage, metastasis, low OS, and sorafenib resistance (Spano et al., 2010; Zhang et al., 2016). The upregulation of Gal-1 in HCC cells is considered the primary source of soluble Gal-1 in TME. Interestingly, emerging reports demonstrate that Gal-1 is also wildly expressed in cancer stromal cells, such as fibroblasts, endothelial cells, T cells, and macrophages (Martinez-Bosch and Navarro, 2020). In response to stimuli from cancer cells or TME, stromal cell-derived Gal-1 can act with a paracrine effect to support cancer development. For example, fibroblast-produced Gal-1 induces migration and invasion of breast cancer or gastric cancer cells by triggering increased matrix metallopeptidase or epithelial-mesenchymal transition (EMT), respectively, (Chong et al., 2016; Zhu et al., 2016). Activated endothelial cells induced by prostate cancer cells can upregulate Gal-1 expression to reduce T-cell transendothelial migration (He and Baum, 2006). The contribution of TAM-derived Gal-1 in TME has not been explored before. Our animal studies showed that Gal-1 deficient TAMs attenuated HCC tumor growth and reduced the serum level of Gal-1 in HCC-bearing mice. In line with previous reports as discussed above, our data suggest that TAM-derived Gal-1 plays a role in facilitating HCC growth. Of note, Gal-1-carrying TAMs significantly increased the serum level of Gal-1 in HCC-bearing mice, indicating that TAM-derived Gal-1 may contribute to systemic effects of Gal-1, for example, the systemic immunosuppression induced by circulating Gal-1 in tumor-bearing mice (Dalotto-Moreno et al., 2013).

Since Gal-1 lacks a specific signal sequence, the secretion of Gal-1 is suggested to be via an unconventional pathway. Indeed, our findings here revealed that Gal-1 is released from HCC-stimulated macrophages by a TLR2/ROS-mediated secretory autophagy. Our data echoed a recent study showing that a TLR-dependent PI3K signal activates Gal-1 secretion on ovarian cancer cells (Park et al., 2017). This indicates that TLR-regulated Gal-1 secretion is not limited to TAMs. The PI3K signal has recently been linked to regulating autophagy in response to changing ROS levels. PI3K/mTOR signal acts as an autophagy inhibitor under moderate ROS levels but becomes an autophagy promoter in response to high ROS levels (Kma and Baruah, 2021). In addition, the increase of cellular ROS levels triggers the unconventional secretion of antioxidants (Cruz-Garcia et al., 2020). Based on these observations, the interplay between PI3K and ROS under TLR signaling may be crucial for inducing Gal-1 secretion in TAMs. On the other hand, identifying factors in triggering TLR2-mediated Gal-1 secretion from TAMs could be the therapeutic target for HCC. Our recent study identified an HCC-derived TLR2 ligand called high mobility group box 1 (HMGB1), which is responsible for inducing TLR2-mediated autophagy in TAMs (Shiau et al., 2020). Interestingly, in our pilot test, recombinant HMGB1 protein could induce Gla-1 release from BMDMs via TLR2 (Supplementary Figure 3), suggesting that HMGB1 is a potential HCC-derived factor to stimulate Gal-1 secretion from TAMs.

It is still an unsolved question of how autophagosome-engulfed cargos are delineated to secretory or degradative autophagy. However, the different subsets of MVB, as well as Rab and SNARE proteins, might be essential to mediate secretory autophagy. A previous study demonstrated that the level of cholesterol in MVBs might play a role in secretory and degradative autophagy. Cholesterol-rich MVBs were found to facilitate secretory autophagy, while cholesterol-poor MVBs were targeted for degradative autophagy (Mobius et al., 2002). Of note, TLR signal-induced increase of intracellular ROS can promote cholesterol synthesis (Li et al., 2013; Tsoi et al., 2017). Thus, TLR2-mediated increase of ROS in TAMs might lead to generating cholesterol-rich MVBs for Gal-1 secretion via autophagy. On the other hand, specific recruitment of Rab8a and Sec22b in secretory autophagy while Rab8b and Stx17 are recruited in degradative autophagy has been observed (Padmanabhan and Manjithaya, 2020). Here, we found the MVB/Rab11/VAMP7 axis but not Stx17 participating in secretory autophagy of Gal-1 in TAMs. The critical roles of Rab8a and Sec22b in TAM-secreted Gal-1 are worthy of further investigation.

In cohorts of patients with HCC, the high Gal-1 expression in HCC tumor cells has been largely proven to be a poor prognostic factor after tumor resection, significantly associated with poor OS (Wu et al., 2012, 2018; Shao et al., 2020). However, the Gal-1 expression in TAMs has seldom been investigated, and, to the best of our knowledge, our study is the first to demonstrate its clinical significance in HCC. Interestingly, our data demonstrated that the HCC with low Gal-1 expression in TAMs had larger tumor size, higher stage, more tumor recurrences, and deaths, indicating that Gal-1 plays a different role between HCC tumor cells and TAMs. In addition, the serum Gal-1 levels reflected a similar scenario; patients with high serum Gal-1 levels (over 250 ng/ml) had high infiltration of Gal-1^–^LC3^+^ TAMs in their HCCs (Figure 5B). Since the HCC patients with dominant Gal-1^–^ LC3^+^ TAMs had the worst OS and PFS in our cohort, serum Gal-1 levels may be a helpful surrogate to determine the dominant phenotypes of TAMs and further be an adverse prognostic indicator to classify patients at high risk. Moreover, various Gal-1 inhibitors with immunomodulatory, anti-migratory, anti-invasive, and anti-angiogenic activities may be potential agents in cancer therapy (Sethi et al., 2021).

In conclusion, this study provides evidence to show that HCC cells can stimulate macrophages to secrete Gal-1 via TLR2-mediated secretory autophagy. The autophagosome carrying Gal-1 is secreted out in HCC-associated macrophages with the help of MVBs, Rab11, and VAMP7. This autophagy-regulated Gal-1 secretion in TAMs facilitates HCC growth in mice and correlates with the poor prognosis of HCC patients. Our findings highlight the pathological role of Gal-1 secretion by secretory autophagy in TAM-associated HCC progression.

## Data Availability Statement

The original contributions presented in the study are included in the article/Supplementary Material. Further inquiries can be directed to the corresponding author.

## Ethics Statement

The studies involving human participants were reviewed and approved by the institutional review board approved the study design and patient recruitment (CYCH-IRB No. 106-030 and CYCH-IRB No. 104079, Ditmanson Medical Foundation Chia-Yi Christian Hospital, Taiwan). Written informed consent for participation was not required for this study in accordance with the national legislation and the institutional requirements. The animal study was reviewed and approved by the animal study was approved by the Committee on the Ethics of Animal Experiments of National Cheng Kung University (Permit Number: 107130).

## Author Contributions

C-PC: conceptualization, study design, data curation, editing, supervision, and project administration. GD and C-CC: writing the original draft. C-PC and C-CC: funding acquisition. C-CC: resources, editing, statistical analysis, data curation, and visualization. GD: methodology, formal analysis, investigation, and visualization. Y-CC, H-WT, and H-CC: resources and editing. Y-LC, P-JT, W-TK, NT, and Y-SL: investigation and editing. All authors read and approved the final manuscript.

## Conflict of Interest

The authors declare that the research was conducted in the absence of any commercial or financial relationships that could be construed as a potential conflict of interest.

## Publisher’s Note

All claims expressed in this article are solely those of the authors and do not necessarily represent those of their affiliated organizations, or those of the publisher, the editors and the reviewers. Any product that may be evaluated in this article, or claim that may be made by its manufacturer, is not guaranteed or endorsed by the publisher.
