# Supplementary material for: Autophagy Drives Galectin-1 Secretion From Tumor-Associated Macrophages Facilitating Hepatocellular Carcinoma Progression
Source: Front Cell Dev Biol. 2021 Sep 6;9:741820. doi: 10.3389/fcell.2021.741820 (PMC8450461; doi:10.3389/fcell.2021.741820)
Supplement: Supplementary file 1 [file Data_Sheet_1.PDF]

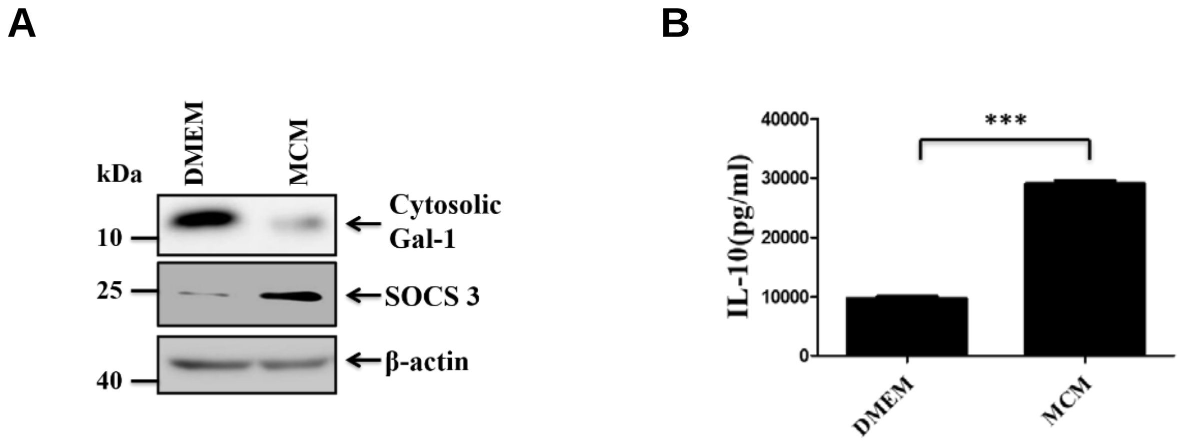

Supplementary Figure 1. MCM stimulates M2-phenotype of BMDMs. BMDMs were treated with MCM for 24 hours to collect the cell lysates and supernatants. **(A)** The protein expression of Gal-1, SOCS3 and β-actin was determined by Western blotting. **(B)** The level of IL-10 in supernatants was detected by ELISA. Statistical analyses between DMEM and MCM were evaluated using Student's t-tests. Statistical significance was defined as ***$p < 0.01$.

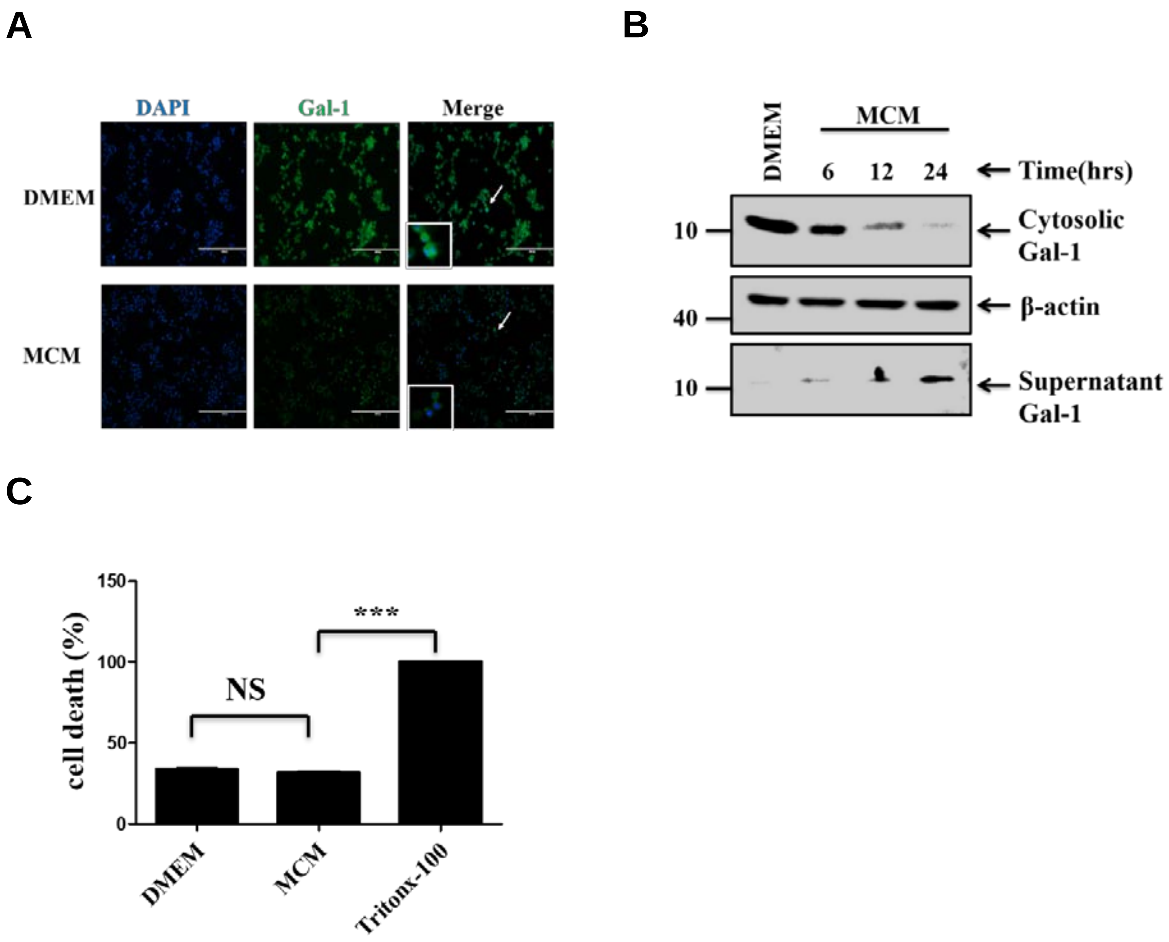

Supplementary Figure 2. MCM induces Gal-1 secretion in RAW264.7 cells. **(A-B)** RAW 264.7 cells were treated with MCM for 24 hours **(A)** or indicated time **(B)**. The expression of Gal-1 was determined by IFA in **(A)** or Western blotting in **(B)**. **(C)** BMEMs were treated with MCM or Triton X-100 (1%) for 24 hours. The cell death was determined by LDH assay. Statistical significance was defined as ***$p < 0.01$.

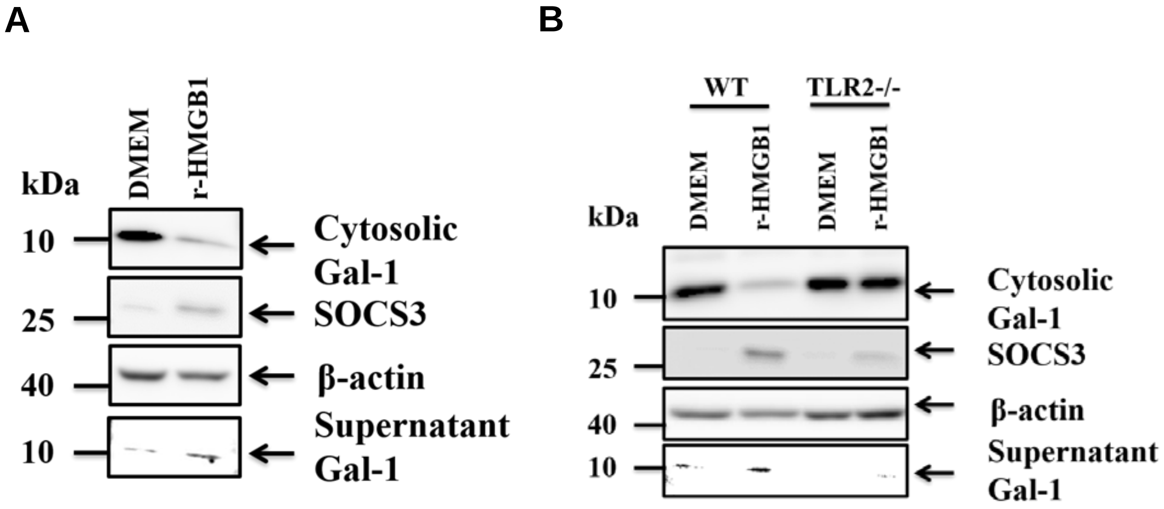

Supplementary Figure 3. Recombinant HMGB1 triggers Gal-1 release via TLR2 in BMDMs. **(A)** BMDMs were treated with recombinant HMGB1 (5 μg/ml) for 24 hours. The expression of cytosolic Gal-1, SOCS3, β-actin and supernatant Gal-1 was determined by Western blotting. **(B)** WT or TLR2-/- BMDMs were treated with recombinant HMGB1 (5 μg/ml) for 24 hours. The expression of cytosolic Gal-1, SOCS3, β-actin and supernatant Gal-1 was determined by Western blotting.

Supplementary Table 1. Baseline Characteristics of Liver Cancer Patients

	Total	$Gal\text{-}1^{-}$ $LC3^{-}$	$Gal\text{-}1^{-}$ $LC3^{+}$	$Gal\text{-}1^{+}$ $LC3^{-}$	$Gal\text{-}1^{+}$ $LC3^{+}$	*P* value
Patient number	N=93	N=7	N=35	N=33	N=18	
Diagnosis Age, Mean(SD)	63.06(10.46)	62.57(16.41)	62.47(10.91)	61.36(9.34)	67.49(8.27)	0.128
Gender						
Female, N(%)	27(29.03)	2(28.57)	8(22.86)	9(27.27)	8(44.44)	0.446
Male, N(%)	66(70.97)	5(71.43)	27(77.14)	24(72.73)	10(55.56)	
HBV						
Negative, N(%)	58(62.37)	5(71.43)	17(48.57)	24(72.73)	12(66.67)	0.211
Positive, N(%)	35(37.63)	2(28.57)	18(51.43)	9(27.27)	6(33.33)	
HCV						
Negative, N(%)	37(39.78)	4(57.14)	16(45.71)	11(33.33)	6(33.33)	0.525
Positive, N(%)	56(60.22)	3(42.86)	19(54.29)	22(66.67)	12(66.67)	
AFP						
<=200ng/mL	73(78.49)	4(57.14)	30(85.71)	23(69.70)	16(88.89)	0.122
>200ng/mL	20(21.51)	3(42.86)	5(14.29)	10(30.30)	2(11.11)	
HAI score, Mean(SD)	9.24(3.53)	7.43(4.31)	9.60(3.09)	9.48(3.41)	8.78(4.22)	0.410
Cirrhosis						
No, N(%)	41(44.09)	5(71.43)	16(45.71)	13(39.39)	7(38.89)	0.490
Yes, N(%)	52(55.91)	2(28.57)	19(54.29)	20(60.61)	11(61.11)	
Tumor size(mm)						
Mean(SD)	42.85(25.61)	71.14(41.90)	51.74(25.96)	43.06(17.06)	30.67(15.31)	<0.001
Stage						

1, N(%)	20(21.51)	0(0.00)	4(11.43)	10(30.30)	6(33.33)	0.005
2, N(%)	45(48.39)	2(28.57)	12(34.29)	20(60.61)	11(61.11)	
3, N(%)	25(26.88)	5(71.43)	16(45.71)	3(9.09)	1(5.56)	
4, N(%)	3(3.22)	0(0.00)	3(8.57)	0(0.00)	0(0.00)	
Recurrence						
No, N(%)	28(30.11)	2(28.57)	5(14.29)	12(36.36)	9(50.00)	0.033
Yes, N(%)	65(69.89)	5(71.43)	30(85.71)	21(63.64)	9(50.00)	
Death						
No, N(%)	51(54.84)	4(57.14)	10(28.57)	23(69.70)	14(77.78)	<0.001
Yes, N(%)	42(45.16)	3(42.86)	25(71.43)	10(30.30)	4(22.22)	

Supplementary Table 2. Cox Proportional Hazard model for HCC groups with different Gal-1 and LC3 expression in TAMs.

		Crude HR		Adjusted HR	
		HR(95% CI)	P Value	HR(95% CI)	P Value
Death					
	Gal-1 $^{+}$ LC3 $^{-}$	Ref.		Ref.	
	Gal-1 $^{-}$ LC3 $^{-}$	1.88(0.52,6.86)	0.340	1.45(0.35,6.05)	0.613
	Gal-1^{-} LC3 $^{+}$	3.82(1.82,8.01)	<0.001	2.77(1.18,6.48)	0.019
	Gal-1 $^{+}$ LC3 $^{+}$	0.73(0.23,2.34)	0.598	0.71(0.22,2.29)	0.567
Recurrence or Death					
	Gal-1 $^{+}$ LC3 $^{-}$	Ref.		Ref.	
	Gal-1 $^{-}$ LC3 $^{-}$	1.86(0.70,4.95)	0.212	2.98(0.94,9.51)	0.065
	Gal-1^{-} LC3 $^{+}$	3.83(2.16,6.78)	<0.001	4.38(2.22,8.67)	<0.001
	Gal-1 $^{+}$ LC3 $^{+}$	0.78(0.37,1.66)	0.517	0.85(0.40,1.83)	0.678

*Adjusted for age, gender, tumor size, stage. HR= hazard ratio.
